# Effect of antioxidants on knee cartilage and bone in healthy, middle-aged subjects: a cross-sectional study

**DOI:** 10.1186/ar2225

**Published:** 2007-07-06

**Authors:** Yuanyuan Wang, Allison M Hodge, Anita E Wluka, Dallas R English, Graham G Giles, Richard O'Sullivan, Andrew Forbes, Flavia M Cicuttini

**Affiliations:** 1 Department of Epidemiology and Preventive Medicine, Monash University, Central and Eastern Clinical School, Alfred Hospital, Melbourne, VIC 3004, Australia; 2 Cancer Epidemiology Centre, The Cancer Council of Victoria, Carlton, VIC 3053, Australia; 3 Baker Heart Research Institute, Commercial Road, Melbourne, VIC 3004, Australia; 4 School of Population Health, The University of Melbourne, Australia; 5 MRI Unit, Mayne Health Diagnostic Imaging Group, Epworth Hospital, Richmond, VIC 3121, Australia

## Abstract

The aim of the present study is to examine the effect of dietary antioxidants on knee structure in a cohort of healthy, middle-aged subjects with no clinical knee osteoarthritis.

Two hundred and ninety-three healthy adults (mean age = 58.0 years, standard deviation = 5.5) without knee pain or knee injury were selected from an existing community-based cohort. The intake of antioxidant vitamins and food sources by these individuals was estimated from a food frequency questionnaire at baseline. The cartilage volume, bone area, cartilage defects and bone marrow lesions were assessed approximately 10 years later using magnetic resonance imaging.

In multivariate analyses, higher vitamin C intake was associated with a reduced risk of bone marrow lesions (odds ratio = 0.50, 95% confidence interval (CI) = 0.29–0.87, *P *= 0.01) and with a reduction in the tibial plateau bone area (β = -35.5, 95% CI = -68.8 to -2.3, *P *= 0.04). There was an inverse association between fruit intake and the tibial plateau bone area (β = -27.8, 95% CI = -54.9 to -0.7, *P *= 0.04) and between fruit intake and the risk of bone marrow lesions (odds ratio = 0.72, 95% CI = 0.52–0.99, *P *= 0.05). Neither fruit intake nor vitamin C intake was significantly associated with the cartilage volume or cartilage defects. Lutein and zeaxanthin intake was associated with a decreased risk of cartilage defects (odds ratio = 0.71, 95% CI = 0.51–0.99, *P *= 0.04), and vitamin E intake tended to be positively associated with the tibial plateau bone area (β = 33.7, 95% CI = -3.1 to 70.4, *P *= 0.07) only after adjusting for vitamin C intake. The β-cryptoxanthin intake was inversely associated with the tibial plateau bone area after adjusting for vitamin E intake (β = -33.2, 95% CI = -63.1 to -3.4, *P *= 0.03). Intake of vegetables and other carotenoids was not significantly associated with cartilage or bone measures.

The present study suggests a beneficial effect of fruit consumption and vitamin C intake as they are associated with a reduction in bone size and the number of bone marrow lesions, both of which are important in the pathogenesis of knee osteoarthritis. While our findings need to be confirmed by longitudinal studies, they highlight the potential of the diet to modify the risk of osteoarthritis.

## Introduction

Osteoarthritis (OA) is a disease affecting the whole joint, including the articular cartilage, bone and soft tissues. OA is the most common form of joint disease and cause of musculoskeletal disability in the elderly [1]. Nutrients and dietary supplements have been shown to be effective at relieving the symptoms of OA, and some may have a role in influencing the course of OA [2]. There is growing recognition of the importance of nutritional factors in the maintenance of bone and joint health [3].

Reactive oxygen species, which are generated by cells within joints and cause oxidative damage to various macromolecules, have been shown to play a role in the pathogenesis of OA [4]. Vitamin C, vitamin E, and carotenoids are excellent antioxidants that protect cells from damage by oxidants, and whose blood concentrations are primarily determined by dietary intake [5,6]. These antioxidants may have a beneficial effect on joint health. The Framingham OA Cohort Study suggested that dietary vitamin C, vitamin E, and β-carotene reduced the risk of progression of knee OA [7]. In contrast, we showed no effect of supplementary vitamin E or dietary antioxidant vitamins on symptoms and progression of disease in knee OA during 2 years [8,9]. All studies to date, however, have been carried out in patients with OA. No studies have examined the effect of antioxidants on knee cartilage and bone in healthy subjects, prior to symptomatic disease.

Magnetic resonance imaging (MRI) has good tissue contrast and good anatomical resolution [10], and therefore allows a noninvasive examination of the joint structure in predisease or in the early stages of OA. MRI can visualize the joint structure directly [11] and has been recognized as a valid, accurate, and reproducible tool to measure the articular cartilage volume [11,12], cartilage defects [13,14], the tibial plateau bone area [12,15], and bone marrow lesions [16,17], which have been shown to have important roles in OA [12-14,17]. A major strength of examining these structural features is that we can examine the state of knee from a normal condition through to the predisease and early disease.

In the present study, we utilize dietary data from the Melbourne Collaborative Cohort Study (MCCS) [18] to examine the association of antioxidants and foods rich in these antioxidants with knee cartilage and bone measures in healthy, community-based, middle-aged men and women with no clinical knee OA.

## Patients and methods

### Participants

The study was conducted within the MCCS, which is a prospective cohort study of 41,528 residents of Melbourne, Australia aged between 27 and 75 years (99.3% were aged 40–69 years) at recruitment, which occurred between 1990 and 1994. The study's aim was to examine the role of lifestyle factors in the risk of cancer and heart disease [18].

Participants were recruited via the electoral rolls (registration to vote is compulsory for adults in Australia), advertisements, and community announcements in the local media (for example, television, radio, and newspapers). Participants for this current study were recruited from MCCS. As our intent was to investigate subjects with no significant current or past knee disease, individuals were excluded if they had had any of the following: a clinical diagnosis of knee OA as defined by American College of Rheumatology criteria [19]; knee pain lasting for >24 hours in the past 5 years; a previous knee injury requiring nonweight-bearing treatment for >24 hours or surgery (including arthroscopy); a malignancy; the participant was unable to complete the study (for example, proposed relocation); or the participant had a history of any form of arthritis diagnosed by a medical practitioner. A further exclusion criterion was a contraindication to MRI including pacemaker, metal sutures, the presence of shrapnel or iron filings in the eye, or claustrophobia.

We invited subjects who fulfilled our inclusion criteria and attended the first year of round-three follow-up of the MCCS, which commenced in 2003. We used quota sampling whereby recruitment ceased when our target sample of approximately 300 subjects was achieved. By the end of 2004, 297 eligible subjects were recruited into the current study. The study was approved by The Cancer Council Victoria's Human Research Ethics Committee and by the Standing Committee on Ethics in Research Involving Humans of Monash University. All participants gave written informed consent.

### Anthropometric and dietary data

Extensive information was collected at MCCS baseline (1990–1994) using questionnaires and physical measurements. Questionnaires covered demographic data and diet – via a 121-item food frequency questionnaire developed from a study of weighed food records [20]. Nutrient intakes were calculated from the food frequency questionnaire using Australian food composition data [21], and using the US Department of Agriculture database for carotenoids (α-carotene, β-carotene, β-cryptoxanthin, lutein and zeaxanthin, lycopene) [22]. All nutrient intakes reflect those from food only without supplements. Fruits and vegetables are important food sources of vitamin C, vitamin E, and carotenoids [6,23]; they were therefore chosen as potentially influential foods, and their intakes were assessed from the food frequency questionnaire [20]. Participant weight was measured using electronic scales with bulky clothing removed. Their height was measured using a stadiometer with shoes removed. The body mass index (weight/height^2 ^(kg/m^2^)) was calculated.

### MRI and measurement of cartilage volume, bone area, cartilage defects, and bone marrow lesions

During 2003–2004, each subject had an MRI scan performed on the dominant knee (defined as the lower limb the subject used to step off when walking). Knees were imaged on a 1.5-T whole-body magnetic resonance unit (Philips 1.5 Tesla Intera; Philips Medical Systems, Eindhoven, The Netherlands) using a commercial transmit–receive extremity coil, with sagittal T_1_-weighted fat-suppressed three-dimensional gradient recall acquisition and coronal T_2_-weighted fat-saturated acquisition as previously described [12,16].

The tibial cartilage volume was determined by image processing on an independent workstation using Osiris software (Geneva, Switzerland) as previously described [11,12]. The measurement was performed by two independent trained observers. One observer measured all subjects, and the other observer carried out cross-checks; that is, measured randomly selected subjects, choosing one out of five subjects, in a blinded fashion. The coefficients of variation for cartilage volume measures were 2.1% for the medial tibial and 2.2% for the lateral tibial cartilage [12].

The tibial plateau cross-sectional area was used as a measure of tibial bone size from images reformatted in the axial plane using Osiris software, as previously described [12,15]. Using this technique, osteophytes, if present, are not included in the area of interest. The measurement was performed by two independent trained observers. One observer measured all subjects, and the other observer carried out cross-checks; that is, measured randomly selected subjects, choosing one out of five subjects, in a blinded fashion. The coefficients of variation for the medial tibial and the lateral tibial plateau areas were 2.3% and 2.4%, respectively [12].

Cartilage defects were graded on the magnetic resonance images with a classification system previously described [13,14], for the medial and lateral tibial and femoral cartilages. The measurement was carried out by a single trained observer, who measured all images in duplicate on separate occasions. A cartilage defect was identified as present if there was any irregularity on the cartilage surface or the cartilage bottom with a loss of cartilage thickness on at least two consecutive slices at any site of that compartment. The intraobserver reliability and interobserver reliability assessed in 50 magnetic resonance images (expressed as the intraclass correlation coefficient) were 0.90 and 0.90 for the medial tibiofemoral compartment, and were 0.89 and 0.85 for the lateral tibiofemoral compartment, respectively [14].

Bone marrow lesions were defined as areas of increased signal intensity adjacent to subcortical bone in either the distal femur or the proximal tibia [16,17]. A lesion was identified as present if it appeared on two or more adjacent slices in either tibiofemoral compartment [16,17]. Two trained observers, who were blinded to the characteristics of subjects, together assessed the presence of lesions for each subject. The reproducibility for determination of bone marrow lesions was assessed by the same method as used to measure bone marrow lesions, using 60 randomly selected knee MRI scans (κ = 0.88, *P *< 0.001) from a different population measured on two occasions.

### Statistical analyses

With 297 subjects, the present study had 80% power to show a correlation as low as 0.15 between the various risk factors and the knee cartilage volume (α error = 0.05, two-sided significance), thus explaining up to 2.2% of the variance of cartilage volume. The present study also had 80% power to detect an odds ratio of 1.4 for cartilage defects or of 1.7 for bone marrow lesions, associated with a one-standard-deviation increase in a continuous predictor (α error = 0.05, two-sided significance).

The outcomes were the tibial cartilage volume, the tibial plateau bone area, and the presence of tibiofemoral cartilage defects and bone marrow lesions. The first two outcomes were initially assessed for normality before being regressed against intakes of food and nutrients. They showed a normal distribution, and thus linear regression was used. The presence/absence of tibiofemoral cartilage defects and bone marrow lesions were dichotomous outcomes, and thus logistic regression was used.

Participants with self-reported total energy intakes in the top or bottom 1% of the sex-specific distributions were excluded. Multivariate regression models were constructed to explore the relationship between food or antioxidant intake and the knee structure elements, adjusting for potential confounders of age, gender, body mass index, and energy intake. Food intakes were divided into quartiles and assigned the median value for the quartile; hence the odds ratios reflect the odds associated with an increase of one serving per day in intake. Dietary antioxidants were standardized so that the coefficients represent the effect of a one-standard-deviation increment in intake. *P *< 0.05 was considered statistically significant. All analyses were performed using the SPSS statistical package (standard version 14.0; SPSS, Chicago, IL, USA).

## Results

Two hundred and ninety-seven participants entered the study. After excluding four subjects with energy intakes in the top or bottom 1% of the sex-specific distributions, there were 293 participants (63% females, aged 58.0 ± 5.5 years, body mass index of 25.2 ± 3.8 kg/m^2^) remaining in the analyses (Table 1). There were no significant differences between this population and the original MCCS population, which has the following profile: 61% females, aged 57.8 ± 3.0 years, and body mass index of 25.7 ± 3.8 kg/m^2^. There were no significant differences in dietary intakes or other health-related behaviors such as smoking: 60% subjects never smoked in this population versus 57% in the MCCS population.

**Table 1:** Characteristics of study participants

	Total (*n *= 293)
Age when magnetic resonance imaging performed (years)	58.0 (5.5)
Female (*n *(%))	184 (63%)
Variables in 1990–1994	
Body mass index (kg/m^2^)	25.2 (3.8)
Vegetables (times/day) (median (interquartile range))	5.0 (4.0–7.0)
Fruits (times/day) (median (interquartile range))	4.0 (2.0–5.0)
Energy from dietary intake (kJ/day)	9,364.9 (3,067.5)
Vitamin C (mg/day)	218.3 (107.3)
Vitamin E (mg/day)	8.3 (3.4)
Carotenoids	
α-Carotene (μg/day)	1,387.0 (697.3)
β-Carotene (μg/day)	5,821.1 (2,507.0)
β-Cryptoxanthin (μg/day)	421.3 (348.1)
Lutein and zeaxanthin (μg/day)	1,822.5 (908.9)
Lycopene (μg/day)	7,782.6 (5,067.5)
Variables in 2003–2004	
Tibial cartilage volume (mm^3^)	3,731 (1,118)
Presence of any tibiofemoral cartilage defects (*n *(%))	181 (62%)
Tibial plateau bone area (mm^2^)	3,302 (475)
Presence of any tibiofemoral bone marrow lesions (*n *(%))	39 (13%)

Data presented as the mean (standard deviation), unless stated otherwise.

### Relationship between vitamin C and vitamin E intake and knee cartilage and bone measures

After adjusting for potential confounders, the vitamin C intake was inversely associated with the tibial plateau bone area (β = -35.5, 95% confidence interval (CI) = -68.8 to -2.3, *P *= 0.04) and with the presence of bone marrow lesions (odds ratio = 0.50, 95% CI = 0.29–0.87, *P *= 0.01). The vitamin C intake was not significantly associated with tibial cartilage volume or the presence of cartilage defects. There was no significant association between vitamin E intake and knee cartilage or bone measures (Table 2). When intakes of vitamin C and vitamin E were added to the regression model simultaneously, most of the findings did not change (results not shown) – except that the vitamin E intake tended to be positively associated with the tibial plateau bone area (β = 33.7, 95% CI = -3.1 to 70.4, *P *= 0.07) while the vitamin C intake was still significantly negatively associated with the tibial plateau bone area (β = -40.2, 95% CI = -73.7 to -6.7, *P *= 0.02).

**Table 2:** Relationship between vitamin C and vitamin E intake and knee structures

	Univariate analysis		Multivariate analysis	
	
	Regression coefficient (odds ratio (95% confidence interval))	*P *value	Regression coefficient (odds ratio (95% confidence interval))	*P *value
Vitamin C				
Cartilage volume^a^	-41.9 (-173.2 to 89.4)	0.53	-60.8 (-147.9 to 26.3)	0.17
Cartilage defects^b^	1.03 (0.81–1.31)	0.82	1.02 (0.76–1.36)	0.90
Bone area^c^	-9.3 (-65.1 to 46.5)	0.74	-35.5 (-68.8 to -2.3)	0.04
Bone marrow lesions^d^	0.63 (0.40–0.99)	0.05	0.50 (0.29–0.87)	0.01
Vitamin E				
Cartilage volume	186.5 (58.4–314.5)	0.004	57.3 (-37.8 to 152.4)	0.24
Cartilage defects	0.96 (0.76–1.22)	0.74	1.00 (0.72–1.33)	0.89
Bone area	73.8 (19.3–128.2)	0.01	27.0 (-9.6 to 63.6)	0.15
Bone marrow lesions	1.10 (0.80–1.50)	0.56	1.10 (0.73–1.66)	0.66

^a^Change in tibial cartilage volume (mm^3^) per standard-deviation increase in vitamin C/vitamin E intake before and after adjusting for energy intake, age, gender, body mass index, and tibial plateau bone area.

^b^Odds ratio of tibiofemoral cartilage defects being present per standard-deviation increase in vitamin C/vitamin E intake before and after adjusting for energy intake, age, gender, body mass index, and tibial cartilage volume.

^c^Change in tibial plateau bone area (mm^2^) per standard-deviation increase in vitamin C/vitamin E intake before and after adjusting for energy intake, age, gender, and body mass index.

^d^Odds ratio of tibiofemoral bone marrow lesions being present per standard-deviation increase in vitamin C/vitamin E intake before and after adjusting for energy intake, age, gender, and body mass index.

### Relationship between carotenoid intake and knee cartilage and bone measures

After adjusting for potential confounders, the β-cryptoxanthin intake tended to be associated with a decreased tibial plateau bone area (β = -25.5, 95% CI = -54.4 to 3.5, *P *= 0.09) and with the presence of bone marrow lesions (odds ratio = 0.64, 95% CI = 0.38–1.07, *P *= 0.09). These marginal significances disappeared after vitamin C intake was added to the models. The β-cryptoxanthin intake, however, was inversely associated with the tibial plateau bone area after adjusting for vitamin E intake (β = -33.2, 95% CI = -63.1 to -3.4, *P *= 0.03). The intake of lutein and zeaxanthin was associated with a decreased presence of cartilage defects only after vitamin C intake was added to the model (odds ratio = 0.71, 95% CI = 0.51–0.99, *P *= 0.04). There was no significant association between the intake of other carotenoids and knee cartilage or bone measures in the multivariate analyses (Table 3).

**Table 3:** Relationship between carotenoid intake and knee structures

	Univariate analysis		Multivariate analysis	
	
	Regression coefficient (odds ratio (95% confidence interval))	*P *value	Regression coefficient (odds ratio (95% confidence interval))	*P *value
α-Carotene				
Cartilage volume^a^	-65.6 (-194.8 to 63.6)	0.32	-1.3 (-77.0 to 74.4)	0.97
Cartilage defects^b^	1.02 (0.81–1.30)	0.84	1.01 (0.79–1.30)	0.91
Bone area^c^	-32.8 (-87.6 to 22.0)	0.24	-3.8 (-33.1 to 25.4)	0.80
Bone marrow lesions^d^	1.15 (0.83–1.58)	0.40	1.13 (0.80–1.61)	0.48
β-Carotene				
Cartilage volume	-43.3 (-172.9 to 86.3)	0.51	2.5 (-75.6 to 80.6)	0.95
Cartilage defects	0.97 (0.76–1.22)	0.77	0.95 (0.74–1.23)	0.72
Bone area	-26.3 (-81.3 to 28.6)	0.35	-12.5 (-42.7 to 17.6)	0.42
Bone marrow lesions	0.97 (0.68–1.36)	0.85	0.92 (0.62–1.34)	0.65
β-Cryptoxanthin				
Cartilage volume	-27.2 (-155.9 to 101.5)	0.68	-32.5 (-108.1 to 43.1)	0.40
Cartilage defects	0.87 (0.69–1.10)	0.25	0.89 (0.70–1.14)	0.36
Bone area	-8.4 (-63.1 to 46.3)	0.76	-25.5 (-54.4 to 3.5)	0.09
Bone marrow lesions	0.64 (0.39–1.06)	0.08	0.64 (0.38–1.07)	0.09
Lutein and zeaxanthin				
Cartilage volume	0.8 (-128.4 to 130.0)	0.99	-2.4 (-79.2 to 74.5)	0.95
Cartilage defects	0.83 (0.65–1.05)	0.13	0.83 (0.64–1.07)	0.14
Bone area	-5.8 (-60.7 to 49.0)	0.84	-4.3 (-34.0 to 25.5)	0.78
Bone marrow lesions	0.68 (0.44–1.06)	0.09	0.68 (0.43–1.08)	0.10
Lycopene				
Cartilage volume	86.8 (-41.6 to 215.2)	0.19	-9.9 (-87.0 to 67.3)	0.80
Cartilage defects	0.98 (0.78–1.24)	0.89	1.04 (0.81–1.35)	0.75
Bone area	36.6 (-17.9 to 91.2)	0.19	-9.7 (-39.5 to 20.1)	0.52
Bone marrow lesions	0.75 (0.49–1.15)	0.19	0.78 (0.51–1.21)	0.27

^a^Change in tibial cartilage volume (mm^3^) per standard-deviation increase in the respective carotenoid intake before and after adjusting for energy intake, age, gender, body mass index, and tibial plateau bone area.

^b^Odds ratio of tibiofemoral cartilage defects being present per standard-deviation increase in the respective carotenoid intake before and after adjusting for energy intake, age, gender, body mass index, and tibial cartilage volume.

^c^Change in tibial plateau bone area (mm^2^) per standard-deviation increase in the respective carotenoid intake before and after adjusting for energy intake, age, gender, and body mass index.

^d^Odds ratio of tibiofemoral bone marrow lesions being present per standard-deviation increase in the respective carotenoid intake before and after adjusting for energy intake, age, gender, and body mass index.

### Relationship between fruit and vegetable intake and knee cartilage and bone measures

After adjusting for potential confounders, fruit intake was inversely associated with the tibial plateau bone area (β = -27.8, 95% CI = -54.9 to -0.7, *P *= 0.04) and with the presence of bone marrow lesions (odds ratio = 0.72, 95% CI = 0.52–0.99, *P *= 0.05). Fruit intake was not significantly associated with the tibial cartilage volume or with the presence of cartilage defects. Vegetable intake was not significantly associated with knee cartilage or bone measures (Table 4).

**Table 4:** Relationship between fruit and vegetable intake and knee structures

	Univariate analysis		Multivariate analysis	
	
	Regression coefficient (odds ratio (95% confidence interval))	*P *value	Regression coefficient (odds ratio (95% confidence interval))	*P *value
Fruit				
Cartilage volume^a^	-154.2 (-269.9 to -38.5)	0.01	-55.7 (-126.4 to 15.0)	0.12
Cartilage defects^b^	1.08 (0.87–1.34)	0.46	1.06 (0.84–1.34)	0.62
Bone area^c^	-58.0 (-107.3 to -8.7)	0.02	-27.8 (-54.9 to -0.7)	0.04
Bone marrow lesions^d^	0.75 (0.55–1.03)	0.07	0.72 (0.52–0.99)	0.05
Vegetables				
Cartilage volume	-120.4 (-241.5 to 0.6)	0.05	20.6 (-52.0 to 93.1)	0.58
Cartilage defects	0.99 (0.79–1.24)	0.93	0.93 (0.73–1.17)	0.52
Bone area	-66.5 (-117.7 to -15.4)	0.01	-2.1 (-30.2 to 26.0)	0.88
Bone marrow lesions	1.05 (0.77–1.45)	0.76	1.01 (0.72–1.42)	0.97

^a^Change in tibial cartilage volume (mm^3^) per serving per day increase in fruit/vegetables intake before and after adjusting for energy intake, age, gender, body mass index, and tibial plateau bone area.

^b^Odds ratio of tibiofemoral cartilage defects being present per serving per day increase in fruit/vegetables intake before and after adjusting for energy intake, age, gender, body mass index, and tibial cartilage volume.

^c^Change in tibial plateau bone area (mm^2^) per serving per day increase in fruit/vegetables intake before and after adjusting for energy intake, age, gender, and body mass index.

^d^Odds ratio of tibiofemoral bone marrow lesions being present per serving per day increase in fruit/vegetables intake before and after adjusting for energy intake, age, gender, and body mass index.

Adding lifestyle factors such as physical activity, education level, smoking, and alcohol consumption to the multivariate models did not alter the results (data not shown).

## Discussion

In this population of healthy, middle-aged people with no clinical knee OA, vitamin C intake was inversely associated with the tibial plateau bone area and with the presence of bone marrow lesions, both of which are important in the pathogenesis of knee OA. Consistent with fruit being an important source of vitamin C, fruit intake was also found to be inversely associated with the tibial plateau bone area and with the presence of bone marrow lesions. These data suggest a beneficial effect of vitamin C and fruit on bone structure. The lutein and zeaxanthin intake was associated with a decreased risk of cartilage defects independent of vitamin C intake, and β-cryptoxanthin intake was associated with decreased tibial plateau bone area independent of vitamin E intake, suggesting a beneficial effect of these carotenoids on knee cartilage and bone. The vitamin E intake, however, tended to be positively associated with the tibial plateau bone area independent of vitamin C intake, which is a negative effect on the bone.

There is conflicting evidence on the role of vitamin C and vitamin E with regard to the risk of knee OA. The Framingham OA Cohort Study showed that vitamin C intake reduced the risk of progression of knee OA, and that vitamin E intake reduced the risk of OA progression in men only [7]. In contrast, ascorbic acid supplementation worsened spontaneous OA in a guinea pig model [24], and we previously showed no effect of supplementary vitamin E on the change in knee cartilage volume in a randomized placebo-controlled trial during 2 years in subjects with knee OA [8]. All data available to date have been in patients with established OA. No previous studies have examined the relationship between vitamin C and vitamin E intake and knee structure in healthy subjects. In our current study performed on healthy subjects free of clinical knee OA, we found a negative association between vitamin C intake and the tibial plateau bone area and the presence of bone marrow lesions. This suggests a protective effect of vitamin C on the risk of knee OA since previous studies have suggested that an increase in bone size is a very early response of the knee to known risk factors for knee OA [25,26]. For example, changes of bone expansion are seen in response to increased adductor moment [25] and obesity [26] even before changes are seen in cartilage. Moreover, the bone area is increased in patients with OA compared with those without OA [27], and the area increases over time in those with OA [15]. This cannot be explained by osteophytes which were not included in the bone area measurements. In addition, bone marrow lesions have been shown to be associated with pain and progressive joint space loss in knee OA [16,17]. These findings may explain the mechanism by which vitamin C effects the previously reported reduction in the risk of knee OA [7]. Vitamin E intake, however, was shown to be associated with an increased tibial plateau bone area, which is thought to be an adverse finding in terms of knee structure in OA [15].

The evidence regarding the effect of carotenoids on the risk of knee OA is limited. The Framingham OA Cohort Study showed that β-carotene intake reduced the risk of progression of knee OA [7]. A case–control study performed by De Roos and colleagues, however, found that those in the highest tertile of serum lutein or β-cryptoxanthin were less likely to have knee OA than controls, and those in the highest tertile of serum β-carotene or zeaxanthin were more likely to have knee OA [28]. In contrast, our study found that the lutein and zeaxanthin intake was associated with a decreased risk of cartilage defects, and that β-cryptoxanthin intake was associated with a decreased tibial plateau bone area – both suggesting a beneficial effect on the knee. There was no significant association between any other carotenoids and knee cartilage or bone. This discrepancy may partly be explained by the different methods used to measure exposure. The Framingham OA Cohort Study and our study assessed dietary intake rather than serum levels. Although serum measures reflect the dietary intake of the carotenoids, they also reflect differences in interindividual absorption and metabolism. Moreover, when examining the effect of carotenoid intake on the knee, we used a more sensitive method of assessing knee structure than the Framingham study and De Roos and colleagues' study, which used radiographic assessment of the knee joint [7,28].

The present study has a number of limitations. We were able to measure dietary nutrients in a valid fashion [29]. A single measure of nutrient intakes 10 years earlier, however, was used as the exposure measure in our study, which may not reflect more recent and perhaps relevant intake, if intervening illness or other life changes affected the intake. While not all studies have shown dietary stability in adults, there is some evidence that the nutrient intake is relatively stable and tends to be more stable with increasing age [30,31]. We have no way of knowing what the situation is in the MCCS cohort. Longitudinal studies have suggested that individuals may move toward a more healthy diet over time [32,33]. The participants in our study are likely to represent the more healthy and health conscious of all those who were initially recruited into the MCCS, since they were selected from those who took part in the first year of the follow-up. They may have already adopted a healthy diet and thus be less likely to change in this direction. While selection bias towards healthier subjects may have affected the estimates of nutrient intake and knee structure measures, it is unlikely that this would modify the relationships between nutrient intakes and knee structure. Although nutritional data collected 10 years earlier may have resulted in some misclassification of exposure, such misclassification is likely to be nondifferential in relation to knee structure, since only subjects with no history of knee symptoms or injury were included. Nondifferential misclassification tends to underestimate the strength of any observed associations. The prospective design is also a potential strength of our study since a substantial period of time has elapsed between the ascertainment of exposure to nutritional factors and the development of outcomes (cartilage and bone measures). Another limitation of this study is that no information on dietary supplements was available, and we were therefore unable to adjust the effect of these supplements in the statistical analyses.

Articular cartilage and bone health is dependent upon the regular provision of nutrients, and it has been suggested that diets deficient in nutrients may lead to arthropathy [3]. The effect of foods and nutrients on knee structure is likely to be complex. Our study suggests that the direct effect of vitamin C is on bone rather than on cartilage. Although vitamin C and vitamin E are known potent antioxidants, given that different effects of vitamin C and vitamin E were found on the bone area in the present study, the mechanism of action in this situation may not be via an antioxidant effect. Vitamin C is a cofactor in the hydroxylation of lysine and proline, and therefore is required in the cross-linking of collagen fibrils in bone. Vitamin C stimulates alkaline phosphatase activity, a marker for osteoblast formation. Several studies have reported a beneficial effect of vitamin C intake on the bone mineral density [34,35]. A higher bone mineral density is associated with greater rigidity and strength of the bone. Bone may therefore expand less in relation to factors such as increased loading on the bone. This may provide an explanation of the association of higher vitamin C intake with decreased bone area and the risk of bone marrow lesions. The emerging evidence of structural change in OA and pre-OA suggests that bony changes occur early and that cartilage defects predate changes in the cartilage volume, which in turn occur before any radiological change is evident. This continuum acknowledges that bone plays an important role in early OA. Recent work has suggested that the well described risk factors for OA, including obesity and the knee adduction moment, may act through an effect on tibial bone before any effect on cartilage occurs [25,26]. The enlargement of the tibial plateau bone may attenuate the tibial cartilage, and this attenuation may play a role in the pathogenesis of OA [15].

## Conclusion

The present study suggests a beneficial effect of vitamin C intake on the reduction in bone size and the number of bone marrow lesions, both of which are important in the pathogenesis of knee OA. Our study also suggests benefits for bone health from fruit consumption, consistent with fruit being an important source of vitamin C. These observations support the dietary recommendation for eating more fruit. While our findings need to be confirmed by larger longitudinal studies, they do highlight the potential of diet to modify the risk of OA.

## Abbreviations

CI = confidence interval; MCCS = Melbourne Collaborative Cohort Study; MRI = magnetic resonance imaging; OA = osteoarthritis.

## Competing interests

The authors declare that they have no competing interests.

## Authors' contributions

FMC and YW participated in the design of the study. DRE, GGG, and RO participated in the acquisition of data. YW carried out the measurement of knee cartilage and bone structure, performed the statistical analysis and interpretation of data, and drafted the manuscript. AF provided statistical support. AMH, AEW, FMC, DRE, and GGG participated in the analysis and interpretation of data, and reviewed the manuscript. All authors read and approved the final manuscript.
